# Biohydrogen Production from Waste Black Cumin (Nigella Sativa) Extract Liquid

**DOI:** 10.3390/bioengineering11030282

**Published:** 2024-03-16

**Authors:** Nesrin Dursun, Hakki Gülşen

**Affiliations:** 1 Department of Environmental Health, Ardahan University, 75002 Ardahan, Turkey; 2 Department of Construction Technologies, Ardahan University, 75002 Ardahan, Turkey; 3 Department of Environmental Engineering, Harran University, 63190 Sanliurfa, Turkey; hgulsen@harran.edu.tr

**Keywords:** biohydrogen production, black cumin extract liquid, mixed culture, bioreactor

## Abstract

Hydrogen creates water during combustion. Therefore, it is expected to be the most promising environmentally friendly energy alternative in the coming years. This study used extract liquid obtained from the waste nigella sativa generated by the black cumin oil industry. The performance of biological hydrogen manufacturing via dark fermentation was investigated in the fluidized bed reactor (FBR) and completely stirred tank reactor (CSTR) under the operation conditions of pH 5.0, 4.0, and 6.0 and a hydraulic retention time (HRT) of 36 and 24 h. The performance of hydrogen manufacturing was determined to be good under an organic loading ratio (OLR) of 6.66 g.nigella sativa extract/L and pH 4.0. According to these conditions, the maximum amount of hydrogen in CSTR and FBR was found to be 20.8 and 7.6 mL H_2_/day, respectively. The operating process of the reactors displayed that a reduction in HRT augmented biohydrogen manufacturing. The work that used mixed culture found that the dominant microbial population at pH 4.0 involved *Hydrogenimonas thermophila*, *Sulfurospirillum carboxydovorans*, *Sulfurospirillum cavolei*, *Sulfurospirillum alkalitolerans*, and *Thiofractor thiocaminus*. No research on waste black cumin extract was found in biohydrogen studies, and it was determined that this substrate source is applicable for biological hydrogen manufacturing.

## 1. Introduction

The worldwide trend of increasing production and consumption significantly affects global emissions, which are the main source of global climate change [1]. The subsequent demand for energy has led to increased use of fossil fuels, and the impact of greenhouse gases on global climate change has become significant. Therefore, exploring sustainable fuel alternatives has become quite critical. In this context, hydrogen obtained from biofuels is the lightest and most ample element in the universe. The hydrogen content is approximately 0.14% on the earth’s surface and 0.07% in the atmosphere. A total of 1 L air mass is stated as about 1.2 g and 1 L hydrogen mass as 0.09 g [2]. Hydrogen production is carried out using various methods. In this regard, it was reported that hydrogen is produced through steam reforming of organic compounds [3], biomass gasification, steam gasification, pyrolysis, and so on [4,5,6]. Additionally, hydrogen can be produced biologically. Biological hydrogen production was reported to be affected by environmental and operational factors [7]. The main environmental and operational factors that are important in this context are presented below. *Substrate Sources*: Typically, waste or wastewater with high carbohydrate content is used for hydrogen production. Some substrate sources such as waste and wastewater from the food industry (food and beverage industry), animal waste, agricultural waste, milk, starch processing, sugar, fruit processing waste, etc., can be listed as usable sources for hydrogen gas production [8]. *pH*: It is accepted as one of the factors affecting the result of biohydrogen fermentation since it significantly influences the activity of dominant species according to the studies using mixed culture as well as hydrogenase enzyme(s). Depending on the composition of the feed solution and the microbial population, the optimal pH value can vary widely [9]. Studies determined a typical pH range of 4.0–7.0. Mota et al. [10] conducted a study that contradicted with the up-to-date published literature data that reports powerful inhibition by dark fermentation at pH values lesser than 4.0. That study found stable and long-term hydrogen production at an average pH of 2.7. That study opened a new research area towards a more sustainable and applicable technology in hydrogen production via biological fermentation. *Hydrogen Producing Bacteria*: Conversion of the substrate source to hydrogen gas via fermentation is typically carried out through a row of complex biochemical/metabolic reactions and by various specific types of bacteria such as *Clostridia*, *Escherichia coli*, *Enterobacter*, and *Citrobacter*. In addition to pure cultures, it was used for biological hydrogen manufacturing in a variety of mixed microflora and cultures [8]. In the work by Ren et al. [11], acid, heat, alkali, and repeated aeration pretreatments were applied to the mixed culture in order to increase hydrogen-producing bacteria in mixed microbial cultures. The studies using mixed culture typically involve pretreatments. *Temperature*: It is vital in the operation of continuous-feed biohydrogen reactors. Depending on the type of raw material used in the reactors, they can be operated in mesophilic or thermophilic temperature ranges [9]. *Bioreactor Types and Operation*: Bioreactors used in biohydrogen production can be reserved into two groups—(i) batch bioreactors; (ii) continuous-feed bioreactors—according to the continuity in the feed flow. According to the process selection in continuous bioreactors, two types of bioreactors were developed: immobilized bioreactors (anaerobic fixed bed reactor (AFBR), upflow anaerobic sludge bed reactor (UASBR), and fluidized bed reactor (FBR)) and suspended growth bioreactors (anaerobic membrane reactor and completely stirred tank reactor). Completely stirred bioreactors have often been used for operating systems larger than these bioreactor types. For simple operation and effective control, batch reactors have been used [8,12,13]. *Hydraulic retention time (HRT)*: It is one of the significant reactor check parameters that is affected by the biohydrogen manufacturing rate and operations in bioreactors [9]. The operating conditions of reactors also vary depending on whether the substrate source is liquid or solid.

The potential of various liquid or liquefied raw materials for biohydrogen production has been examined in various studies. These studies used specific bacteria or mixed consortia. In this regard, some specific bacteria have been reported to be *Clostridium thermocellum* [14], *Clostridium beijerinckii* [15], and *Clostridium saccharoperbutylacetonicum* N1-4 [16,17]. Mixed bacteria, on the other hand, have been used more frequently than specific bacteria in biohydrogen production research, mainly because they are easily available. In studies using mixed bacteria, the mixed bacteria were subjected to thermal pretreatment to enrich hydrogen-producing, spore-forming bacteria and to eliminate or enhance the activity of hydrogen-consuming microorganisms. In some studies using mixed bacteria, thermal pretreatment was applied to activated sludge mixture at 105 °C for 30 min [18], to anaerobic sludge at 105 °C for 30 min [19], to landfill leachate sludge at 65 °C for 30 min [20], and to sludge from a food and paper waste compost source at 80 °C for 30 min [21]. Studies conducted in this context have reported that the following conditions support hydrogen production: in a study where dairy product wastewater was used, the optimum temperature, pH, and hydraulic residence time (HRT) were 55 °C, 6.5, and 6 h, respectively [22]; in a study where brewery effluent was used, the optimum pH, temperature, and HRT were 6.5, 55 °C, and 18 h [23], respectively; in a batch study using coconut milk wastewater, an initial pH value of 6.5 and 35 ± 2 °C were used [24]; in a batch study using slaughterhouse sludge and vegetal wastewater, a pH value of 6.5 and 50 °C were used [25]; in a batch study where mozzarella cheese and pecorino cheese wastewater was examined, a pH value in the range of 6.5–7.5 and 39 ± 1 °C were used [18]; in a batch study using glucose, fructose, sucrose, and xylose, 37 °C and an initial pH value of 5.5 were used [26]; in a study examining lactate wastewater originating from the food processing industry, 45 °C and a pH value of 7.5 were used [19]; in a batch study examining dairy product wastewater, an initial pH of 6 and 37 °C were used [20]; and, finally, in a batch study examining citrus processing industry wastewater, 37 °C and a pH value of 5.5 were sued [27].

Nigella sativa generated by the black cumin oil industry can also be included in the group of waste or wastewater sources with high carbohydrate content, which is one of the most significant factors affecting biohydrogen manufacturing. In this context, we aimed to evaluate the extract liquid obtained by extracting waste black cumin obtained from a black cumin oil producer in terms of biohydrogen production potential. The waste composition was determined as 35% protein, 15.5% fat, 43.5% carbohydrate, and 6% ash. The components of black cumin per 100 g were reported by some researchers [28,29,30] as 20.02~24.05 g of protein, 21.67~37.33 g of oil, and 30.5~39.04 g of carbohydrate, as well as ash damp and fiber. The literature contains various studies examining biological hydrogen production through dark fermentation using mixed bacteria, but no research on biological hydrogen production from black cumin extract liquid was found. In addition, to the best of the authors’ knowledge, there is no study in biohydrogen production research on the study of the extract liquid obtained via decoction pretreatment of waste as a substrate source in continuous-feed bioreactors.

This research used liquid extracted from waste obtained from the black cumin industry and aimed to determine the biological hydrogen manufacturing performance via dark fermentation in CSTR and FBR at different pH values and OLRs. In the study, mixed inoculum was used and microbial community structures were examined for each operating condition and at various pH values.

## 2. Materials and Methods

### 2.1. Bioreactors and Operation

**Figure 1:**
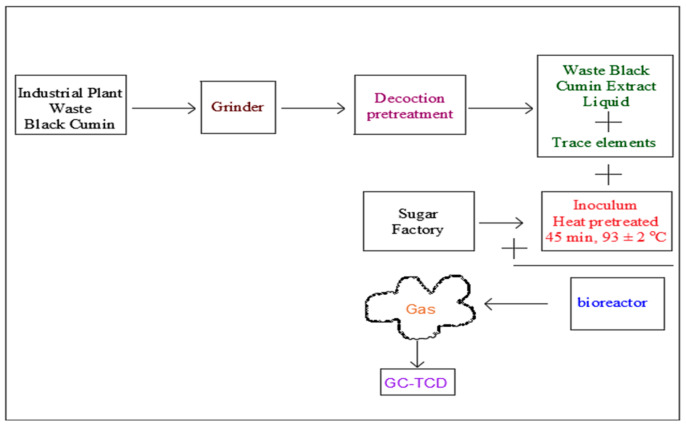
Schematic illustration of the study.

Sludge was obtained from the UASBR of the biologic wastewater treatment facility of the sugar factory operating in the province of Malatya to use as the inoculum. Just before the sludge was added to the bioreactor, heat pretreatment (93 ± 2 °C for 45 min) was applied to the sludge in order to increment the activity of hydrogen-generating microorganisms and inhibit/completely destroy methanogenic microorganisms. Waste black cumin was crushed in a grinder and then decoction pretreatment was applied to the waste. As trace elements, KH_2_PO_4_, NH_4_Cl, CaCl_2_·2H_2_O, NiCl_2_.6H_2_O, MgSO_4_·7H_2_O, ZnCl_2_, Na_2_MoO_4_·2H_2_O, CuCl_2_·H_2_O, FeCl_3_·6H_2_O, MnCl_2_·4H_2_O, CoCl_2_·6H_2_O, and H_3_BO_3_ were used in the amounts studied by Fang et al. [31]. A schematic illustration of the study is presented in Figure 1.

In a temperature-controlled dark room at 35 ± 2 °C, the bioreactors were veiled with aluminum foil before operation in order to prohibit the development of phototrophic microorganisms. Nutrient solution was kept in a fridge operated at +4 °C to prohibit biologic activity. The solution was prepared daily, and N_2_ gas was applied to the feed solution for 25 min to deoxygenate it. The reactor was fed immediately after this step.

Below, detailed information on the operating circumstances of bioreactors is given.

#### 2.1.1. Completely Stirred Tank Reactor (CSTR) Fed with Waste Black Cumin Extract Liquid

A magnetic stirrer was added to a laboratory-scale CSTR made of glass material with an empty bed volume of 3000 mL and a total volume of 5000 mL, and full mixing was carried out using the magnetic stirrer. Another 5000 mL bioreactor was added to this bioreactor to prevent the bioreactor contents from separating in the system and to collect wastewater. To accelerate and enhance biofilm formation, activated carbon (1.5–2.0 mm) granules were added to the CSTR. In addition, the thermally pretreated inoculum was added to the bioreactor. In the study, decoction pretreatment was applied to waste black cumin and the extracted liquid was used. According to this method, distilled water should be used. However, tap water was used instead of distilled water in this study. Tap water was added to black cumin, and a heat treatment was applied at 95 ± 5 °C for 30 min. After the heat treatment, the extraction process was completed by filtering through the filter paper. After extraction, a liquid fraction (black cumin extract), which was wealthy in dissoluble carbohydrates, and a solid fraction (residue) were acquired. The bioreactor was nourished with the solution drawn up by supplementing the trace elements in the amounts examined by Fang et al. [31] to the extract liquid obtained via the extraction process of black cumin. The pH (1 M NaOH and 1 M HCI) was adjusted before feeding the reactor with the nutrient solution daily—prepared by supplementing the trace elements into the black cumin extract liquid. To inhibit biological activity, the bioreactor was fed with nutrient solution stored in a refrigerator operating at +4 °C. The bioreactor was stirred intermittently for the first 5 days following the installation. Throughout the operation, in the dark room where the warmth was held constant at 35 ± 2 °C, it was perpetual batch fed at a mixing ratio of 160 rpm. In order to prohibit the activated carbon and bacteria contained in the CSTR from leaving the reactor, no stirring occurred during the feeding period. During the reactor operation, mixing was stopped 60 min before the feed time, so that the contents could settle; 5 min after the end of the feed time, the bioreactor was stirred again at a stirring speed of 160 rpm. The operating conditions of the CSTR are displayed in Table 1.

#### 2.1.2. Fluidized Bed Reactor (FBR) Fed with Waste Black Cumin Extract Liquid

A laboratory-scale FBR made of glass material, stuffed with activated carbon (1.5–2.0 mm), with an empty bed bulk of 600 mL and a sum volume of 1000 mL was used. Activated carbon granules were opted for in the reactor in order to accelerate and increase biofilm formation. The bioreactor was veiled with aluminum foil and continuous batch fed in a dark room at 35 ± 2 °C. To inhibit biological activity, the bioreactor was fed with nutrient solution stored in a refrigerator operating at +4 °C. Return was made intermittently in the first 5 days following the bioreactor setup. In order to fluidize the reactor bed, the bioreactor was return-operated. In order to prohibit the bioreactor contents from penetrating the reactor outlet tank or the return pump, no return was performed while feeding but was performed immediately after the feeding. The reactor return rate was applied in the range of 50 to 100 times the feed solution flow ratio. The processes of obtaining black cumin extract liquid, heat treatment of the inoculum, preparation of the feed solution via the addition of trace elements to the black cumin extract liquid, and pH adjustment of the feed solution were similar to their applications in the CSTR. The operating circumstances of the FBR are displayed in Table 2.

#### 2.1.3. Batch Reactors Fed with Waste Black Cumin Extract Liquid

In order to specify the impact of dissimilar OLRs and pH on biohydrogen manufacturing in the CSTR and FBR, sludge samples were taken on the 28th day of the acclimatization phase in these reactors and added to 120 mL bioreactors (serum bottles). The processes of obtaining black cumin extract liquid, preparation of the feed solution via the of addition trace elements to the black cumin extract liquid, and pH adjustment of the feed solution were similarly to their applications in the CSTR. Once the reactors were three-quarters filled, they were capped and covered with aluminum foil. Immediately after the reactor was set up, N_2_ gas was supplied to the reactor for 5 min to remove the oxygen. The bioreactors were placed in a shaker incubator operated at 170 rpm at 35 ± 2 °C in a dark room.

### 2.2. Analytical Methods

Gas samples were taken using glass injectors with a gasproof cock and analyzed using the thermal conductivity detector (TCD) and the GC (Shimadzu Nexis GC-2030, Kyoto, Japan) with capillary colon RT-Msieve 5A (0.53 mmID, 50 µm df) in HUBTAM Laboratory. Helium was used as the bearer gas. Detector, column, and injection warmth values were 230 °C, 35 °C, and 200 °C, respectively. Calibration was performed using high-purity H_2_, CH_4_, and CO_2_ gases. Liquid specimens were prepared for analyses using cellulose acetate syringe filters with 0.45 µm pore dimensions before measurement. At the exit of the bioreactor, samples were taken for organic acid measurement at particular time intervals. These samples were measured on the HPLC (Varian, Palo Alto, CA, USA) device at the METU Chromatography & Fermentation Laboratory. COD was admeasured using the closed reflux method, pursuant to standard methods [32]. PCR-DGGE analyses were performed at Süleyman Demirel University to specify the dominant species at each pH in the FBR and CSTR. For this analysis, the Qiagen QIAamp DNA Stool Mini Kit (Qiagen, Hilden, Germany, cat. no. 51504) was procured first. DNA isolations were performed by applying the kit’s protocol. DNA samples were appreciated in circumstances of concentration A260/280 and A260/230 rates in a Microvolume Spectrophotometer, mySPEC (VWR, Radnor, PA, USA), appliance. Following this process, it was determined that all of the samples were suitable for subsequent analyses. DNA samples were amplified in PCR using primers that amplify the bacterial V3 gene zone. The Bio-Rad T100 PCR (BioRad, Hercules, CA) system was expended during the amplification step. PCR products acquired using primers in the amplification step were utilized in a 2% agarose gel electrophoresis system. It was ensured that the amplification step took place in the samples and that amplification was achieved for the applicable amount of DGGE. Following these procedures, the specimens were charged into DGGE gel and the gel image was analyzed. The PCR products were sequenced and then the microorganism species were determined in the samples using the NCBI Blast program [33].

## 3. Results and Discussion

### 3.1. The Impact of Hydraulic Retention Time (HRT) and pH on Gas Manufacturing in a CSTR

Trace elements were added to the waste black cumin extract liquid to feed the bioreactor. It was actuated for approximately 100 days in the acclimatization phase and four dissimilar operating periods. There is a restricted number of works that are partly similar to biological hydrogen manufacturing in a CSTR. The study by Antonopoulou et al. [34] used sorghum biomass as the organic matter for hydrogen manufacturing and it was processed in a CSTR. That study enquired about the impact of pH on biohydrogen manufacturing from sweet sorghum extract via continuous fermentation. For extraction, ground sorghum stems were used, and the extraction process was completed by stirring at regular intervals at 30 °C. After extraction, a liquid fraction (sorghum extract), which was wealthy in soluble carbohydrates, and a solid fraction (lignocellulosic residue) were acquired. Reactor operation was carried out under mesophilic circumstances in the pH range of 3.5 to 6.5 and at 12 h HRT. Maximum hydrogen efficiency was detected to be 3.5 L H_2_/L reactor/day at pH 5.3. Optimum biohydrogen production was determined to occur in the pH range of 5.3 to 4.7, where butyric acid was identified as the metabolic produce. Lowering the pH worth to 4.6 reduced the butyric acid concentration, leading to a decline in biohydrogen production. When operated at pH 3.5, it was found that hydrogen production stopped.

**Figure 2:**
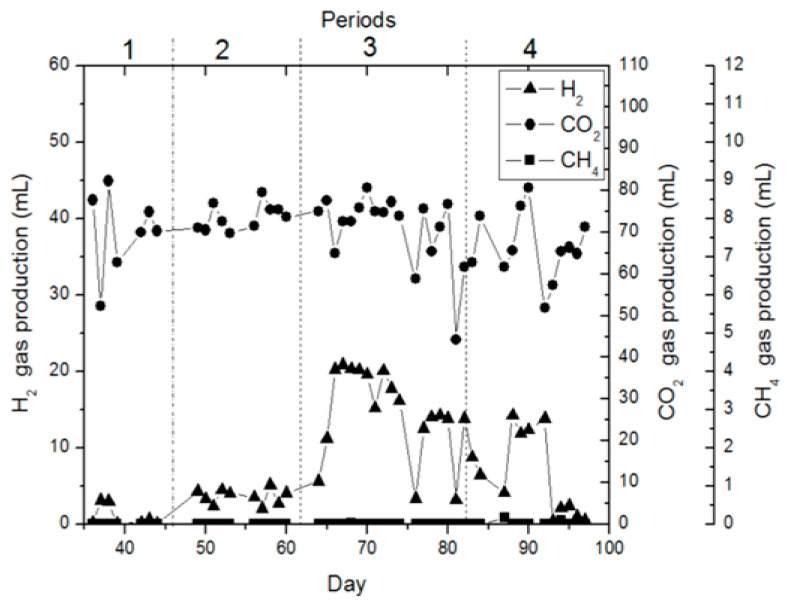
Hydrogen gas production in a CSTR in dissimilar operating periods.

**Figure 3:**
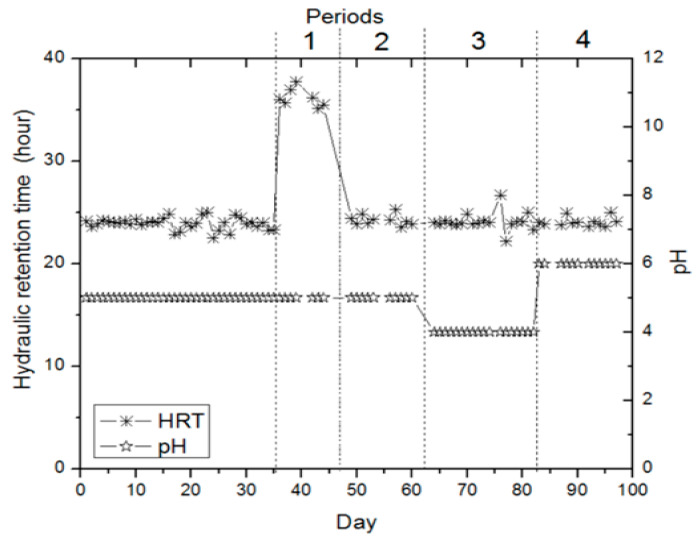
pH and HRT operating conditions in a CSTR.

In CSTR, no gas production was detected to occur in the initial 19 days, but un-balanced gas production was detected in the following days. This acclimatization stage of bacteria lasted for approximately 35 days. During the acclimatization stage, the bioreactor was actuated at an OLR of 4.44 g.nigella sativa extract/L (216.6 mg COD L^−1^), 24 h HRT, and 5.0 pH. Stable gas production and continuous hydrogen fermentation were determined on the 35th day. Also, batch reactors were set up to identify probable impacts before exchanging the operating circumstances in CSTR. In batch reactors, waste black cumin was extracted and trace elements were added to the extract liquid, and hydrogen production potential was examined under different organic loading ratios (2.22 g.nigella sativa extract/L, 4.44 g.nigella sativa extract/L, and 6.66 g.nigella sativa extract/L) and pH values (4.0, 5.0, and 6.0). It was designated that hydrogen production was high in the bioreactors with OLRs of 4.44 g.nigella sativa extract/L and 6.66 g.nigella sativa extract/L. Figure 2 shows the gas production at CSTR for four operating periods. Figure 3 presents the operating conditions during these four gas production periods. In the first period, it was continued as in the acclimatization phase (35 days) with pH 5.0 and the OLR of 4.44 g.nigella sativa extract/L (185.8 mg COD L^-1^), and the reactor operation was carried out with the only change of hydraulic retention time to 36 h. This alteration was intended to expedite gas production in the process, but it was found that the alteration reduced gas production (Figure 2 and Figure 3). It was determined that unstable hydrogen production in CSTR during the initial period was related to the HRT of 36 h, and we decided to operate at 24 h HRT again (Figure 3). Therefore, hydrogen production was found to be low at 36 h of HRT. Similar findings were observed in the work by Salem et al. [35], where continuous biological hydrogen production was examined using sucrose, potato, and bean wastewater in a CSTR. In the study, the effect of 24, 18, and 12 h of HRT on biohydrogen production at a pH value of 5.5 was investigated. It was reported that reducing the HRT to 18 h in wastewater containing sucrose and potato resulted in optimum hydrogen production. In the bean wastewater substrate, 24 h of HRT yielded optimum biohydrogen production. In another work where brewery waste was used as a substrate, the effect of HRT on biohydrogen manufacturing was investigated. In the work, optimum hydrogen production was reported at four diversified pH values (5.0, 5.5, 6.0, and 6.5) with an HRT of 18 h [36]. Contrary to these studies, some studies have reported that an HRT of 8 h or less is suitable for hydrogen manufacturing [37,38,39]. Current research and other studies confirm that optimal HRT may vary depending on the type of waste or wastewater.

Han et al. [40] and Fan et al. [36] reported that butyric and acetic acid production support hydrogen production, while propionic acid production results in less hydrogen production. Table 3 shows organic acid (acetic, propionic, and butyric acid) analyses performed at definite time intervals in CSTR. Similar to the work conducted by Wong et al. [20], in the current work, the main products of organic acids were determined to be acetic and butyric acid. The CSTR was continuously batch fed, and no stirring was performed for about 3 h during the feeding; then, stirring was resumed upon completion of the feeding. In research on continuous biological hydrogen production, attached microbial growth systems and suspended microbial growth systems are generally used. In this context, the CSTR is the most commonly preferred suspended microbial growth system. However, it has been reported that low HRT or high substrate concentration in a CSTR may cause sludge washing. It has also been reported that this situation, in turn, may limit hydrogen production due to operational instability [41,42,43]. Taking this into consideration, reactor operation was carried out in the current study. The COD inlet concentration was measured from the feed tank and the outlet concentration from the exit tank. The continuously stirred tank reactor was actuated at an entry OLR of 185.8 mg COD L^−1^ in the initial period. The exit concentration was determined to be 100.7 mg COD L^−1^ (36–46 days). Under the same conditions, the operating circumstances for the second period (4.44 g.nigella sativa extract/L) (on mean, 191.3 mg COD L^−1^ and pH 5.0) were continued with the only change in the hydraulic retention time to 24 h. In this period, when biohydrogen production became stable with HRT alteration, the maximum biohydrogen production was detected to be 5.1 mL H_2_/day (on the 58th day). In the second term, the mean exit COD concentration was determined to be 109 mg COD L^−1^.

In the third period, the operations were carried out at two different organic loading rates (OLRs). The operating circumstances for the third term included an OLR of 6.66 g.nigella sativa extract/L (mean 275.5 mg COD L^−1^), 24 h HRT, and 4.0 pH between days 64 and 75. With the operating condition of an OLR of 6.66 g.nigella sativa extract/L (on mean, 275.5 mg COD L^−1^) in the third period, the change in pH and organic loading rate compared to the second term resulted in a sharp increment in gas manufacturing performance. At the beginning of this period (between days 64 and 75), 5.6 mL H_2_/day gas production was observed with the change in pH. In the following days (between days 65 and 75), depending on the acclimatization of the bacteria to the environment pH, an average of 18.2 mL H_2_/day hydrogen gas was produced. In the third period, between days 76 and 82, 24 h hydraulic retention time and pH 4.0 were used, and only the organic loading rate was changed to 4.44 g.nigella sativa extract/L (185.8 mg COD L^−1^); an average of 12 mL H_2_/day gas production was determined. In the third term, at OLRs of 6.66 g.nigella sativa extract/L (on mean, 275.5 mg COD L^−1^) and 4.44 g.nigella sativa extract/L (185.8 mg COD L^−1^), the maximum biohydrogen production was detected to be 20.8 mL H_2_/day (67th day) and 14.2 mL H_2_/day (79th day), respectively (Figure 2). In this period, it was determined that biohydrogen manufacturing performance decreased when the OLR was reduced. For the third term, at the OLRs of 6.66 g.nigella sativa extract/L (mean 275.5 mg COD L^−1^) and 4.44 g.nigella sativa extract/L (185.8 mg COD L^−1^), the exit COD concentration was detected to be 147.1 and 96.8 mg COD L^−1^, respectively. In the fourth term, the reactor operation continued at two different organic loading rates (Figure 2). In this period, first of all, the operating conditions were applied between days 83 and 92, as follows: OLR of 6.66 g.nigella sativa extract/L (on mean, 319 mg COD L^−1^), 24 h HRT, and pH 6.0. With the operating condition of an OLR of 6.66 g.nigella sativa extract/L (on mean, 319 mg COD L^−1^), in the fourth period, the change in pH and organic loading rate compared to the third period resulted in no significant change in gas production performance. At the beginning of this term, gas production occurred at around 6.5 mL H_2_/day depending on the acclimatization of bacteria to the environment pH. With this organic loading rate, in the following days (the days between 88 and 92), a biohydrogen production average of 13.1 mL H_2_/day was specified, and the maximum biohydrogen production was found to be 14.2 mL H_2_/day. In the fourth period, between the days 93 and 97, the operating conditions were maintained as 24 h hydraulic retention time and pH 6.0, with the only change in the OLR to 4.44 g.nigella sativa extract/L (185.8 mg COD L^−1^). A gas production average of 2 mL H_2_/day was determined, and the maximum biological hydrogen production was found to be 2.4 mL H_2_/day. In this period, at pH 6.0, the OLR of 4.44 g.nigella sativa extract/L (185.8 mg COD L^−1^) negatively affected the process, and it was determined that hydrogen manufacturing was better with the OLR of 6.66 g.nigella sativa extract/L (on mean, 319 mg COD L^−1^). The mean COD exit concentration for this term was detected to be 163.7 mg COD L^−1^ between days 83 and 92, and 106.8 mg COD L^−1^ between the days 93 and 97. The literature contains various studies on pH and HRT operating parameters. In this context, Silva-Illanes et al. [44] reported that increasing the pH value from 5.5 to 6.0 and reducing the HRT from 12 h to 8 h reduced biohydrogen production. In the current work, bioreactor operation was found to be positive at pH 4.0 with an HRT of 24 h. If these results are evaluated overall, it is confirmed that the optimum operating parameters may vary depending on the type of waste or wastewater, which is consistent with the studies reported in the literature.

### 3.2. The Impact of HRT and pH on Gas Manufacturing in an FBR

Trace elements were added to the waste black cumin extract liquid to feed the bioreactor. The bioreactor was actuated for approximate 100 days including the acclimatization phase and four dissimilar operating periods. The study which is partly similar to biological hydrogen manufacturing using a fluidized bed reactor was conducted by Antonopoulou et al. [45]. In the study, sweet sorghum extract was tested for biohydrogen production at different substrate concentrations in a CSTR. The study was conducted under mesophilic conditions, 12 h HRT, and substrate concentrations in the range of 9.8–20.9 g/L. The maximum biohydrogen production ratio was found to be 2.93 L.H_2_/L.reactor/day at 17.5 g.carbohydrate/L. Butyric acid was identified as the main metabolic product in all stable conditions.

**Figure 4:**
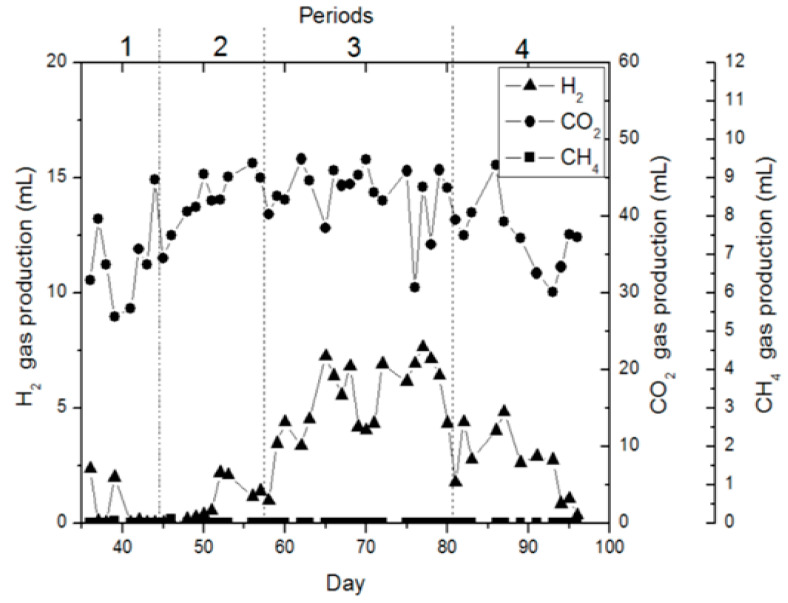
Hydrogen gas production in an FBR in dissimilar operating periods.

**Figure 5:**
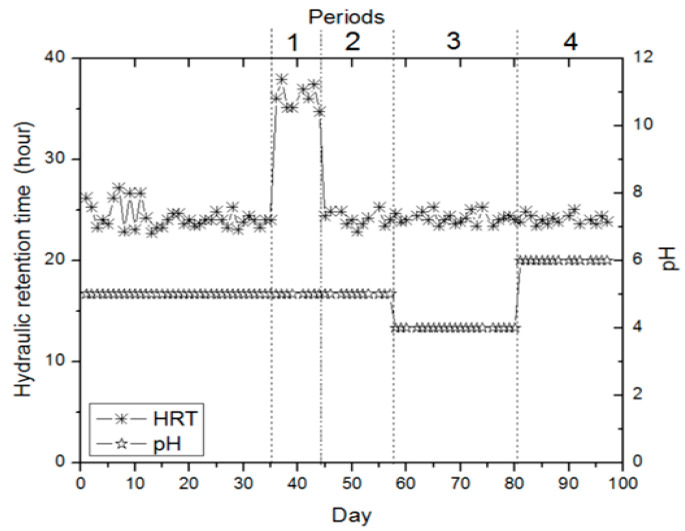
pH and HRT operating conditions in an FBR.

In FBR, the acclimatization phase of the bacteria took 35 days. No gas production was detected in the initial 18 days, but unstable gas production was detected after then. During the acclimatization phase, the bioreactor was actuated at an OLR of 4.44 g.nigella sativa extract/L (216.6 mg COD L^−1^), 24 h HRT, and 5.0 pH. It was found that stable gas production conditions occurred on the 35th day. In addition, evaluations of the batch reactor, which was set up to predict possible effects, were also applied to the FBR. Figure 4 shows the gas production at FBR for four operating periods. Figure 5 presents the operating conditions during these four gas production periods. The first period’s operating conditions were maintained as in the acclimation phase (35 days) with 5.0 pH and an OLR of 4.44 g.nigella sativa extract/L (185.8 mg COD L^−1^), with the only change occurring in the HRT, which was applied as 36 h. It was determined that this alteration considerably limited gas manufacturing (Figure 4 and Figure 5). Since they allow for stirring within the reactor content in terms of operation, CSTR and FBR were used in this study. FBR was continuously batch fed, and no return was performed for about 1 h during feeding; then, return was resumed upon the completion of feeding. During the biohydrogen production studies, the studies were typically conducted in suspended cell systems, which allow for good stirring. Furthermore, it was reported that, when the hydraulic retention time is kept short or when the dilution rates are high, washing-out may occur in the hydrogen-producing microorganisms, and such a situation may limit the production of hydrogen due to operational instability [41]. Another study reported that HRT had a substantial impact on hydrogen manufacturing, and the hydrogen manufacturing enhanced with reduction in the retention time [46]. Similarly, Amorim et al. [47] reported that reducing the HRT from 8 h to 1 h increased biohydrogen production in an anaerobic FBR fed with cassava wastewater. Considering the studies in the literature, after the first period was operated with an HRT of 36 h, the HRT was reduced in all operating periods and the operation was implemented with an HRT of 24 h.

The researchers reported that the overall higher production of butyric and acetic acid, compared to propionic acid production, was crucial for the increase in biohydrogen production. Furthermore, it was also reported that the high production of propionic acid compared to butyric and acetic acid may limit biohydrogen production. Table 4 shows organic acid (acetic, propionic, and butyric acid) analyses performed at definite time intervals in an FBR. The organic acids in the study were generally high in butyric and acetic acid, followed by lower levels of propionic acid. In a similar trend to the results of this study, in their studies, Pachiega et al. [26] found butyric and acetic acid to be high, Wadjeam et al. [48] found butyric acid to be high, and Amorim et al. [47] found acetic acid to be high. An FBR was operated in the first period at an inlet OLR of 185.8 mg COD L^−1^, and the exit concentration was detected to be 116.1 mg COD L^−1^ (36–38 days) and 120 mg COD L^−1^ (39–44 days).

Under the same conditions, the operating circumstances for the second term (4.44 g.nigella sativa extract/L) (on mean, 195.3 mg COD L^−1^ and pH 5.0) were continued with the only change in the hydraulic retention time to 24 h. Researchers report that a reduction in the hydraulic retention time has an increasing impact on hydrogen manufacturing. As shown in Figure 5, hydrogen production did not become steady with the HRT change (day 6), but the steady state started thereafter. During this period, the maximum biohydrogen production was determined to be 2.1 mL H_2_/day (on day 52) (Figure 4). In the second term, the exit COD concentration was detected to be approximately 118.5 mg COD L^−1^. In the third term, the operations were carried out at two different OLRs. The first operating circumstances in the third term, between days 58 and 65, were applied as follows: an OLR of 4.44 g.nigella sativa extract/L (on mean, 208.1 mg COD L^−1^), 24 h HRT, and 4.0 pH. With the operating condition of an OLR of 4.44 g.nigella sativa extract/L (on average 208.1 mg COD L^−1^), in the third period, the only change in pH compared to the second term resulted in a considerable increment in gas manufacturing performance. At the beginning of this period (between days 58 and 65), with the impact of pH alteration on the OLR of 4.44 g.nigella sativa extract/L (on average, 208.1 mg COD L^−1^), gas production of about 4 mL H_2_/day was detected. In the third term, between days 66 and 80, reactor operation was continued with a HRT of 24 h and pH 4.0, with the only alteration occurring in the organic loading rate, which was applied as 6.66 g.nigella sativa extract/L (mean 294.7 mg COD L^−1^); approximately 6.1 mL H_2_/day of hydrogen gas production was detected. In the third term, the maximum biohydrogen production at the OLRs of 4.44 g.nigella sativa extract/L (on mean, 208.1 mg COD L^−1^) and 6.66 g.nigella sativa extract/L (on mean, 294.7 mg COD L^−1^) was found to be 4.5 mL H_2_/day (64th day) and 7.6 mL H_2_/day (78th day), respectively (Figure 4). In this period, it was determined that, when the organic loading ratio was augmented, hydrogen manufacturing performance increased. In the third period, the outlet COD concentration at the OLRs of 4.44 g.nigella sativa extract/L (on mean, 208.1 mg COD L^−1^) and 6.66 g.nigella sativa extract/L (on mean, 294.7 mg COD L^−1^) was found to be approximately 105.1 and 181.4 mg COD L^−1^, respectively. In the fourth term, the operations were carried out at two different organic loading rates. In this period, first of all, the operating conditions were maintained as in the previous period (between days 66 and 80) using the OLR of 6.66 g.nigella sativa extract/L (325.2 mg COD L^−1^) and 24 h HRT, with the only change occurring in pH, which was applied as 6.0 between days 81 and 89. With the pH change, there was a decline in gas manufacturing performance compared to the third period (between days 66 and 80). At the beginning of this period (between days 81 and 89), a gas production average of 3.7 mL H_2_/day gas occurred, depending on the acclimatization of bacteria to the environment pH. In the fourth period, reactor operation was continued with 24-h hydraulic retention time and pH 6.0 between days 90 and 97, with the only change occurring in the organic loading rate, which was applied as 4.44 g.nigella sativa extract/L (188.7 mg COD L^−1^); a gas production average of 2.3 mL H_2_/day was detected. Between days 90 and 97, in the fourth term, it was determined that the OLR of 4.44 g.nigella sativa extract/L (188.7 mg COD L^−1^) at pH 6.0 decreased hydrogen production. In the fourth term, the mean exit COD concentration was detected to be 211.2 mg COD L^−1^ between days 81 and 89 and 113.2 mg COD L^−1^ between days 90 and 97.

Various types of substrate sources were used for biohydrogen manufacturing. In the work by Kim et al. [49], the sewage sludge taken from the primary and secondary sludge thickeners, in the same quantities as food wastes taken from the dining hall, were shredded and stirred into a mixer for biohydrogen manufacturing. Tawfik et al. [50] used a mixture of urban food waste and kitchen waste in varying concentrations. The study by Chen et al. [51] investigated the upgrowth kinetics of bacteria producing hydrogen via darkness fermentation using three dissimilar substrates including dry skimmed milk powder, sucrose, and food refuse. Some of the other studies in the literature used various wastes/wastewater as substrates such as a mixture of urban solid waste and waste from poultry processing plants and slaughterhouses [52]; wastewater containing starch [53]; and wastewater from the dairy industry [54] and molasses [55]. In other studies using certain wastes/wastewater as substrates, the substrates were subjected to various pretreatments to search for the potential of hydrogen manufacturing from specific microorganism species. In such studies, active sludge from a wastewater treatment plant [56], sugarcane pulp waste, [57] and peels of steamed potatoes [58] were used as substrates. It was reported that wastes or wastewater with high carbohydrate content showed good performance in biological hydrogen production. The fact that black cumin waste has a carbohydrate content of 43.5% supports biohydrogen production, together with other operating factors. It has been reported that biomass can be affected by operational factors, such as the mixing of reactor content, the organic loading rate, HRT, etc. [59].

Hydrogen manufacturing in FBRs and CSTRs typically fluctuated throughout the operating period. This may be due to the activity of the dominant species in the mixed culture depending on the pH and the hydrogen gas being the first gas to leave the environment (since it has the lightest molecular weight and the higher transition velocity compared to the other gases) when sampling (using a gas-tight glass syringe). In this study, biohydrogen production was determined at all pH values examined: 5.0, 4.0, and 6.0. However, considering the manufacturing stability in both reactors, the maximum amount of hydrogen was produced at pH 4.0. The reduction of HRT from 36 h to 24 h increased hydrogen production.

### 3.3. Batch Reactors

The biohydrogen manufacturing potential of waste nigella sativa extract liquid at organic loading ratios of 2.22, 4.44, and 6.66 g.nigella sativa extract/L was investigated at pH 5.0 (Table 5). In the bioreactor with 2.22 g.nigella sativa extract/L, the maximum biohydrogen production was detected to be 11.10^−4^ mL between the 21st and 43rd hours; in the bioreactor with 4.44 g.nigella sativa extract/L, the maximum biohydrogen production was detected to be 210.10^−4^ mL at the 19th hour; and, in the bioreactor with 6.66 g.nigella sativa extract/L, the maximum biohydrogen production was detected to be 37.10^−4^ mL between the 15th and 21st hours. After the time of maximum hydrogen production in the reactors, biohydrogen production decreased with the pH alteration and the reduction in the quantity of organic matter.

The biohydrogen manufacturing potential of waste nigella sativa extract liquid at organic loading ratios of 2.22, 4.44. and 6.66 g.nigella sativa extract/L was investigated at pH 4.0 (Table 6). In the bioreactor with 2.22 g.nigella sativa extract/L, the maximum biohydrogen production was determined to be 96.10^−4^ mL between the 15th and 19th hours; in the bioreactor with 6.66 g.nigella sativa extract/L, the maximum biohydrogen production was determined to be 977.10^−4^ mL at the 21st hour. After the time of maximum hydrogen production in the reactors, biohydrogen manufacturing decreased with the pH alteration and the reduction in the quantity of organic matter. In the bioreactor with 4.44 g.nigella sativa extract/L, the maximum biohydrogen production was detected to be 236.10^−4^ mL at the 15th hour and then 162.10^−4^ mL with a sharp drop at the 16th hour. Then, hydrogen production increased to 225.10^−4^ mL at the 20th hour. The cause for re-increase after the reduction may be the effect of the pH alteration on the activity of the dominant species. It is also reported that the pH factor significantly influences the activity of dominant species in complicated culture [9].

The biohydrogen manufacturing potential of waste nigella sativa extract liquid at organic loading ratios of 2.22, 4.44, and 6.66 g.nigella sativa extract/L was investigated at pH 6.0 (Table 7). In the bioreactor with 2.22 g.nigella sativa extract/L, the maximum biohydrogen production was detected to be 107.10^−4^ mL between the 17th and 20th hours; in the bioreactor with 4.44 g.nigella sativa extract/L, the maximum biohydrogen production was detected to be 136.10^−4^ mL at the 17th hour; and, in the bioreactor with 6.66 g.nigella sativa extract/L, the maximum biohydrogen production was detected to be 250.10^−4^ mL between the 17th and 20th hours. After the time of maximum biohydrogen production in the reactors, biohydrogen production reduced with the pH change and the decline in the quantity of organic matter.

Table 8 summarizes some studies investigating hydrogen production according to operating conditions using various types of substrate sources.

### 3.4. Inoculum Content in the CSTR and FBR

Biohydrogen manufacturing is carried out using a mixed culture and specific bacteria. This study used anaerobic sludge of the biologic wastewater treatment facility of a sugar factory after subjecting it to heat treatment. Biologically produced gases in the study were carbon dioxide and hydrogen, and no methane gas was produced in the CSTR or FBR during the study. This supports the effectiveness of the heat pretreatment applied to the sludge (inoculum) to neutralize the methanogens. In one of the studies using mixed cultures, mixed bacterial cultures acquired from a potato field, a soybean field, and a compost pile were heat-treated to inhibit methanogens and richen hydrogen-producing bacteria [64]. In addition, mixed cultures of various types such as sewage microflora [24], sifted soil [65], beach mud [66], anaerobic sludge [67,68,69,70], and aerobic mud [71] were also used in biohydrogen studies. Some studies used specific bacteria. These specific bacteria included *Pseudomonas* species [56], *Clostridium butyricum* [57], and *Thermotoga neapolitana* and *Caldicellulosiruptor saccharolyticus* [58].

Dominant species detected in the CSTR at pH 4.0 and DGGE band intensity of 0.03 included *Sulfurospirillum cavolei*, *Hydrogenimonas thermophila*, *Sulfurospirillum carboxydovorans*, *Sulfurospirillum alkalitolerans*, and *Thiofractor thiocaminus*. At 5.0 and 6.0 pH with a DGGE band intensity of 0.04, the following dominant species were determined: *Erwinia amylovora*, *Brenneria goodwinii*, *CFB group bacteria*, *Salmonella bongori*, and *Enterobacteria*. In the FBR, dominant species were detected at pH 4.0 and 6.0 with a DGGE band intensity of 0.03 and at pH 5.0 with a DGGE band intensity of 0.04. It was found that the dominant species detected in the respective DGGE band intensities in the FBR were identical in the CSTR. Considering previous studies, the dominant bacteria in general are Clostridium sp. In this context, in the study by Liu et al. [72] in which biohydrogen production from sugar wastewater was investigated, as a result of DGGE analysis, *Clostridium* sp., *Clostridium butyricum*, *Pseudomonas* sp., and *Pseudomonas lindanilytica* bacteria were determined to be the dominant bacteria supporting hydrogen manufacturing. In another work, it was reported that, with an optimum HRT of 60 h, the dominant hydrogen-producing bacteria were *Megasphaera* sp., *Clostridium* sp., and *Chloroflexi* sp. [48]. The PCR-DGGE outcomes of this work showed that the bacterial species of *Hydrogenimonas thermophila*, *Sulfurospirillum carboxydovorans*, *Sulfurospirillum cavolei*, *Sulfurospirillum alkalitolerans*, and *Thiofractor thiocaminus* were dominant in the mixed culture with maximum hydrogen production occurring at pH 4.0. These dominant bacterial species produced acetic and butyric acid as the main products at 35 ± 2 °C, using waste black cumin as the extracted liquid substrate. In the biohydrogen studies, no study was found on these dominant species. It is suggested that a mixed culture or specific bacteria dominated by these species can be used in biohydrogen research in the coming years.

When biohydrogen production studies are evaluated, in general, it may not be clearly stated that an increase or decrease in HRT increases or decreases hydrogen production. Biohydrogen studies should consider factors such as nutrients, sludge (inoculum), pretreatment (time and temperature) applied to the sludge, and, particularly, the type of organic material used as a whole for optimum biohydrogen manufacturing.

In the black cumin oil industry, waste black cumin, formed after oil extraction and rich in carbohydrates, can be utilized for biohydrogen production instead of junking, thereby reducing the amount of waste and greenhouse gas production. In this respect, extensive optimization studies on process operating conditions can be carried out to increase biohydrogen efficiency. Furthermore, the efficiency of biohydrogen production can be increased by mixing different types of carbohydrate-rich waste or wastewater. This could make a significant contribution to the circular economy.

## 4. Conclusions

In the present study, waste black cumin generated after black cumin oil production was obtained and the waste black cumin was extracted and the extract liquid was used. The extracted liquid was used after adding trace elements, and the potential for biological hydrogen manufacturing via dark fermentation under different operating conditions was investigated. Biological hydrogen production was extensively studied in CSTR, FBR, and batch bioreactor types under the operation conditions of the organic loading rates of 2.22, 4.44, and 6.66 g.nigella sativa extract/L and pH 5.0, 4.0, and 6.0. In the reactors, biologic hydrogen production was determined under all pH circumstances. The performance of hydrogen production was found to be good under an OLR of 6.66 g.nigella sativa extract/L and pH 4.0. According to these conditions, the maximum biohydrogen production was detected to be 20.8 and 7.6 mL H_2_/day in CSTRs and FBRs, respectively. The dominant microbial population at pH 4.0 was found to include *Hydrogenimonas thermophila*, *Sulfurospirillum carboxydovorans*, *Sulfurospirillum cavolei*, *Sulfurospirillum alkalitolerans*, and *Thiofractor thiocaminus*.

## Figures and Tables

**Table 1:** Operating conditions of the CSTR fed with black cumin waste extract liquid and filled only with activated carbon.

Periods	Days	pH	HRT (h)	Loading * (gr. Black Cumin Extract/L)	Maximum Hydrogen * (mL/Day)
Acclimation phase	0–35	5.0	23.9	2.22–4.44 (1–10)	-
1	36–46	5.0	36.1	4.44 (36)	3.1 (37)
2	47–63	5.0	24.5	4.44 (47)	5.1 (58)
3	64–82	4.0	24.0	6.66–4.44 (64–76)	20.8–14.2 (67–79)
4	83–97	6.0	24.1	6.66–4.44 (83–93)	14.2–2.4 (88–95)

* Values in parenthesis indicate the days when the hydrogen concentration was at its maximum and the days when the organic loading rate was changed.

**Table 2:** Operating conditions of FBR fed with black cumin waste extract liquid and filled only with activated carbon.

Periods	Days	pH	HRT (h)	Loading * (gr. Black Cumin Extract/L)	Maximum Hydrogen * (mL/Day)
Acclimation phase	0–35	5.0	24.1	2.22–4.44 (1–10)	-
1	36–44	5.0	36.1	4.44 (36)	2.3 (36)
2	45–57	5.0	24.1	4.44 (45)	2.1 (52)
3	58–80	4.0	24.1	4.44–6.66 (58–66)	4.5–7.6 (64–78)
4	81–97	6.0	24.0	6.66–4.44 (81–90)	4.8–2.8 (88–92)

* Values in parenthesis indicate the days when the hydrogen concentration was at its maximum and the days when the organic loading rate was changed.

**Table 3:** Organic acid analysis in a CSTR.

pH-Day-Period	Acetic Acid (mg/mL)	Propionic Acid (mg/mL)	Butyric Acid (mg/mL)
5.0-43-1	0.711 ± 0.006	0.025 ± 0.004	0.154 ± 0.001
5.0-50-2	0.861 ± 0.018	0.275 ± 0.017	0.109 ± 0.000
5.0-52-2	0.110 ± 0.007	0.079 ± 0.004	0.233 ± 0.001
4.0-67-3	2.279 ± 0.006	0.063 ± 0.001	1.129 ± 0.006
4.0-82-3	0.902 ± 0.004	0.008 ± 0.002	0.481 ± 0.002
6.0-85-4	0.556 ± 0.004	0.035 ± 0.000	0.034 ± 0.001
6.0-97-4	0.354 ± 0.004	0.024 ± 0.001	0.027 ± 0.001

**Table 4:** Organic acid analysis in an FBR.

pH-Day-Period	Acetic Acid (mg/mL)	Propionic Acid (mg/mL)	Butyric Acid (mg/mL)
5.0-49-2	0.369 ± 0.001	0.013 ± 0.002	0.014 ± 0.001
5.0-52-2	0.140 ± 0.004	0.000	0.012 ± 0.004
4.0-67-3	1.995 ± 0.008	0.057 ± 0.002	0.688 ± 0.000
4.0-80-3	1.881 ± 0.002	0.027 ± 0.001	0.851 ± 0.002
6.0-85-4	0.556 ± 0.002	0.032 ± 0.000	0.037 ± 0.001
6.0-97-4	0.346 ± 0.001	0.012 ± 0.000	0.028 ± 0.001

**Table 5:** Biohydrogen production at pH 5.0 with the use of waste nigella sativa extract liquid at dissimilar OLRs.

Organic Loading Rate (OLR)	Measurement Time (Hour)	H_2_ (10^−4^ mL)	CO_2_ (10^−2^ mL)	CH_4_ (10^−4^ mL)
2.22 g.nigella sativa extract/L	2	0	65	0
	15	7	65	0
	21	11	65	0
	43	11	65	0
	67	4	64	0
4.44 g.nigella sativa extract/L	2	0	64	0
	14	74	65	0
	16	77	65	0
	19	210	65	0
	21	70	65	0
	37	59	65	0
	43	48	65	0
6.66 g.nigella sativa extract/L	2	0	64	0
	15	37	65	0
	21	37	65	0
	39	30	65	0
	64	19	65	0
	86	17	65	0

**Table 6:** Biohydrogen production at pH 4.0 with the use of waste nigella sativa extract liquid at dissimilar OLRs.

Organic Loading Rate (OLR)	Measurement Time (Hour)	H_2_ (10^−4^ mL)	CO_2_ (10^−2^ mL)	CH_4_ (10^−4^ mL)
2.22 g.nigella sativa extract/L	2	0	64	0
	15	96	65	0
	19	96	65	0
	38	77	65	0
	64	26	65	0
	68	26	65	0
4.44 g.nigella sativa extract/L	2	0	65	0
	15	236	65	0
	16	162	65	0
	20	225	65	0
	38	188	65	0
	43	166	65	0
	63	103	65	0
	68	70	64	0
6.66 g.nigella sativa extract/L	2	0	65	0
	16	870	64	0
	21	977	64	0
	39	940	65	0
	64	652	65	0
	86	542	65	0

**Table 7:** Biohydrogen production at pH 6.0 with the use of waste nigella sativa extract liquid at dissimilar OLRs.

Organic Loading Rate (OLR)	Measurement Time (Hour)	H_2_ (10^−4^ mL)	CO_2_ (10^−2^ mL)	CH_4_ (10^−4^ mL)
2.22 g.nigella sativa extract/L	2	0	65	0
	17	107	65	0
	20	107	65	0
	38	70	65	0
	63	4	65	0
4.44 g.nigella sativa extract/L	2	0	65	0
	17	136	65	0
	20	118	65	0
	38	100	65	0
	64	26	65	0
	68	26	65	0
6.66 g.nigella sativa extract/L	2	0	65	0
	17	254	64	0
	20	250	65	0
	38	181	65	0
	61	92	65	0
	85	63	65	0

**Table 8:** Hydrogen production according to operating conditions using various types of substrate sources.

Substrate	Operating Conditions	H_2_ Production	References
Oat straw subjected to enzymatic treatment	Batch reactor, 4.7 g reducing sugars/L, 35 °C, 7.0 pH	110 mL H_2_/L/h	[60]
Oat straw subjected to HCl pretreatment	Batch reactor, 4.7 g reducing sugars/L, 35 °C, 7.0 pH	70 mL H_2_/L/h	[60]
Alcohol industry wastewater	Upflow Anaerobic Sludge Blanket Reactor, OLR 45 g COD/L, 37 °C, 5.5 pH, 0.96 d HRT	125.1 mL H_2_/g COD	[61]
Palm oil mill effluent	Upflow Anaerobic Sludge Blanket (UASB)–Continuous Stirred Tank Reactor (CSTR), OLR 76.5 ± 0.3 g COD/L, 55 °C, 5.5 pH, 9 h HRT	49.22 mL H_2_/g COD	[62]
Rice bran	An amount of 10% total solids in the batch reactor and completion of the remaining volume with water, 37 °C, 7.0 pH	57.65 mL/h	[63]
Rice husk	An amount of 10% total solids in the batch reactor and completion of the remaining volume with water, 37 °C, 7.5 pH	24.29 mL/h	[63]
Rice straw	An amount of 10% total solids in the batch reactor and completion of the remaining volume with water, 37 °C, 7.5 pH	42.51 mL/h	[63]
Rice waste	An amount of 10% total solids in the batch reactor and completion of the remaining volume with water, 37 °C, 7.0 pH	54.73 mL/h	[63]
Nigella sativa extract (liquid)	Completely Stirred Tank Reactor (CSTR), OLR 6.66 g.nigella sativa extract/L (275.5 mg COD L^−1^), 35 ± 2 °C, 4.0 pH, 24 h HRT	20.8 mL H_2_/d	This study
Nigella sativa extract (liquid)	Fluidized Bed Reactor (FBR), OLR 6.66 g.nigella sativa extract/L (294.7 mg COD L^−1^), 35 ± 2 °C, 4.0 pH, 24 h HRT	7.6 mL H_2_/d	This study

## Data Availability

The authors confirm that the data supporting the findings of this study are available within the article.
